# Increased hydrophilic plasma bile acids are correlated with protection from adiposity in skin-specific stearoyl-CoA desaturase-1 deficient mice

**DOI:** 10.1371/journal.pone.0199682

**Published:** 2018-07-02

**Authors:** Sabrina N. Dumas, James M. Ntambi

**Affiliations:** 1 Department of Nutritional Sciences, University of Wisconsin-Madison, Madison, Wisconsin, United States of America; 2 Department of Biochemistry, University of Wisconsin-Madison, Madison, Wisconsin, United States of America; INRA, FRANCE

## Abstract

Stearoyl-CoA desaturase 1 (SCD1) catalyzes the rate limiting step in monounsaturated fatty acid synthesis by inserting a double bond at the delta-9 position of long-chain fatty acids. SCD1 converts stearate (18:0) to oleate (18:1n9) and palmitate (16:0) to palmitoleate (16:1n7), respectively. Mice with global and skin-specific deletion (SKO) of SCD1 exhibit increased whole body energy expenditure and protection against diet-induced adiposity, hepatic steatosis, insulin sensitivity and glucose intolerance. The mechanisms that link cutaneous lipid homeostasis with whole body energy balance are presently unknown. In this study, we reveal that SKO mice demonstrate increased skin surface free cholesterol, decreased circulating total cholesterol and increased taurine-conjugated and hydrophilic bile acids. Tauro-β-muricholic acid, which is a marker of extrahepatic bile acid synthesis, is significantly elevated in SKO plasma. Bile acid signaling through the bile acid-specific receptor TGR5 is known to be protective against obesity and metabolic disease; a phenotype that is similar to SKO mice. We therefore examined TGR5 expression and its downstream mediator, DIO2, in various tissues and found that both TGR5 and DIO2 expression were significantly increased in brown adipose tissue. In sum, we suggest that skin-derived bile acids are involved in the lean and metabolically healthy phenotype of SKO mice.

## Introduction

The development of obesity is multifactorial and can be caused by genetic predisposition, obesogenic environments, social practices, or a combination thereof [1]. Solutions are needed because co-morbidities of obesity include type-2 diabetes, cardiovascular disease, hepatic steatosis, and some types of cancer [1]. Current interventions to fight obesity include any of the following: consumption of more nutritious foods without excess calories, consistent exercise, pharmaceutical appetite suppressants and, as a last resort, gastric bypass surgery [2]. However, these interventions are inadequate for the scope of the obesity epidemic. The most recent estimate indicates that a third of adults in the United States are obese, with increasing incidence among African American and Mexican American women, and all racial groups of men [3]. And for these reasons, alternative methods for weight loss are warranted.

One such alternative may be to target the lipogenic enzyme stearoyl-CoA desaturase 1 (SCD1). This enzyme is responsible for the desaturation of saturated fatty acids stearate and palmitate into oleate and palmitoleate, respectively [4]. Palmitoleate and oleate are the most abundant MUFAs which are substrates for other important lipids such as phospholipids, triglycerides [TG], cholesteryl esters, wax esters, and alkyldiacylglycerols [4]. Additionally, palmitoleate is an important lipokine that serves as a biomarker for metabolic status [5]. Though SCD1 is ubiquitously expressed in both humans and mice, it is highly expressed in metabolically active tissues such as liver, muscle, white and brown adipose tissues, brain, skin and heart [4]. Importantly, SCD1 global knock-out mice (GKO) are insulin sensitive, glucose tolerant, and protected against genetic- and diet-induced weight gain due to their increased energy expenditure [6]. They are also protected against the development of hepatic steatosis and dyslipidemia [6]. Interestingly, liver (LKO) and skin (SKO) specific SCD1 knock-out mice each recapitulate some characteristics of GKO mice, which indicate that skin and liver are important tissues that contribute to the global SCD1-deficient phenotype [7,8]. Because the mechanisms through which skin may affect distal metabolic tissues is not well understood, we set forth to understand this connection. Skin surface lipids of SKO mice are dramatically altered, with levels of free cholesterol and ceramides most dramatically increased [8]. We therefore hypothesized that one of these lipid species might act as a bioactive molecule that mediates the desirable metabolic phenotype of SKO mice.

Cholesterol plays an important role as a key component of skin barrier lipids [9, 10]. But it is also important for cell membrane structure in its free form, and in its esterified form is stored in lipid droplets in the cytoplasm [9]. Cholesterol esters are synthesized by adding a monounsaturated fatty acid to the hydroxyl group of free cholesterol, a reaction that is catalyzed by acyl-CoA:cholesterol acyltransferase (ACAT) [9]. Importantly, oleate, one of the products of SCD1 is the preferred substrate for this enzyme [11]. In SKO mice, there is a deficiency of oleate in the skin and levels of free cholesterol is elevated 2.5-fold [8]. It is also important to note that ACAT and SCD1 are both endoplasmic reticulum resident enzymes that are within proximity, and this facilitates the transfer of de novo synthesized oleate from SCD1 to ACAT [12]. Furthermore, SCD1 deficient mice fed dietary oleate and palmitoleate are not able to synthesize cholesterol esters [11]. Increased levels of free cholesterol are cytotoxic; therefore, it is of utmost importance for the cell to upregulate pathways to dispose of cholesterol [12]. One method of cholesterol reduction is efflux through ATP-binding cassette transporter A1 (ABCA1), which mediates the first step in reverse cholesterol transport [13]. However, cholesterol levels can also be cleared by conversion into other molecules, such as vitamin D, steroid hormones and bile acids [14]. Bile acid signaling through takeda-G-protein-receptor-5 (TGR5) is known to increase energy expenditure and protects against the development of hepatic steatosis and obesity [15]. Because these phenotypes are comparable to SKO mice, we explored the possibility that skin-derived cholesterol is catabolized into bile acids, and these bile acids then signal through TGR5 in distal tissues.

Bile is a well-known digestive agent that emulsifies lipid containing meals to facilitate digestion and absorption [16]. Briefly, large lipid droplets are broken down into smaller lipid droplets by the detergent action of bile acids, which then allows for hydrolysis of lipids by lipase and colipase [17]. Hydrolyzed lipids can then be taken up by enterocytes [17]. The classic biosynthetic pathway for bile acids takes place in the liver, and the bile produced is stored and concentrated in the gallbladder [18]. The rate limiting enzyme of the canonical bile acid synthesis pathway is cytochrome P450 family 7 subfamily A member 1 (cyp7a1) [18]. This pathway is also known as the neutral pathway. However, bile acid synthesis can also be initiated in extrahepatic tissues where cytochrome P450 family 7 subfamily B member 1 (cyp7b1) is the rate limiting enzyme [18]. In this pathway, acidic intermediates are produced, and is appropriately called the acidic pathway. Cyp7b1 is also expressed in the liver and can compensate for loss of cyp7a1 [18]. In this study we propose that excess cutaneous free cholesterol in SKO mice is converted into bile acids, which results in significant changes in the profile of plasma bile acids. These bile acids can then activate TGR5-mediated signaling in brown adipose tissue, contributing to increased energy expenditure and protection from metabolic disease.

## Materials and methods

### Animals and diets

Generation and maintenance of Scd1f/f and SKO mouse lines have been described previously [8]. Mice were maintained on a 12-hour light/dark cycle with free access to water and a high fat diet (Research Diets D12492) for 8 or 9 weeks. Breeder mice were maintained with similar conditions but fed breeder chow diet (Purina 5015). All animals were sacrificed by isoflurane overdose without fasting, and tissues and plasma were quickly removed, snap-frozen in liquid nitrogen, and stored at -80 °C. Skin tissue was harvested whole and includes both the dermis and epidermis. Mouse cages were changed weekly and on the 8th week, feces that accumulated for 7 days were collected for total fat content analysis at the Marshfield Agricultural Research Station. All animal procedures were approved by the Animal Care Research Committee of the University of Wisconsin-Madison. All data presented in this paper are from a new cohort of male HFD-fed Scd1f/f and SKO mice, unless stated otherwise.

### Cell culture

Brown adipocytes were maintained in Dulbecco’s Modified Eagle’s Medium (DMEM) with 10% fetal bovine serum. Upon confluency, the cells were treated with 2.5% SKO or SCD1f/f plasma pooled from 3 mice for 24 hours. SEB1 sebocytes were cultured in DMEM with 10% fetal bovine serum. Upon confluency, SEB1 cells were differentiated into lipid-producing mature sebocytes by treatment with DMEM containing 20μg/mL insulin, 1μM dexamethasone, 0.5mM 3-isobutyl-1-methylxanthine, 10% FBS and 1μM A939572 (an SCD1 inhibitor). Mature sebocytes were then maintained in DMEM supplemented with 20μg/mL insulin, 10% FBS and 1μM A939572. Control and vehicle-controlled cells were incubated for the same period without A939572 or with 1μM dimethyl sulfoxide (DMSO), respectively.

### Quantitative real-time PCR

RNA from all tissues and cells were extracted using TRI reagent. Total RNA was then treated with Turbo DNase (Ambion) before they were reverse transcribed to cDNA using the High Capacity cDNA Reverse Transcription Kit (Applied Biosystems). Relative mRNA expression levels were quantified by cDNA amplification with gene-specific forward and reverse primers and Power SYBR Green PCR Master Mix on an ABI 7500 Fast RT PCR system. Data were normalized to cyclophilin using the ΔΔCt method. Primer sequences are available upon request.

### Immunoblotting

Tissue homogenates and cell lysates were prepared using RIPA buffer (Cell Signaling Technology) with 1 mM PMSF and Protease Inhibitor Cocktail (Calbiochem). Protein concentrations were measured using a BCA Protein Assay Kit (Thermo Scientific). 25 μg protein was separated by 4–20% MP TGX Stain-Free Gel (Bio-Rad) and transferred to a PVDF membrane (Thermo Scientific). Membranes were blocked with 5 percent non-fat dry milk for 1 hour and treated with primary antibody overnight at 4°C. Membranes were then incubated in secondary antibody for 1 hour at room temperature. All primary antibodies were diluted 1:1000 and all secondary antibodies were diluted 1:5000. Primary antibodies used from abcam were: ABCA1 (ab18180), cyp7a1 (ab65596), TGR5 (ab72608), DIO2 (ab77481) UCP1 (ab10983), and oxphos cocktail (ab110412). Cyp7b1 was used from abnova (H00009420-A01). Secondary antibodies used from Santa Cruz Biotechnology, Inc. were: goat (sc2020) and mouse (sc2005), and rabbit (7074s) from Cell Signaling Technology.

### Immunohistochemistry

Fresh skin tissue was frozen in OCT compound and cut into 10-micron sections and stained with either ABCA1 (ab18180) or Cyp7b1 (24889-1-AP) at a 1:250 dilution. Images were obtained using a Nikon Intensilight Fluorescence Microscope.

### Cholesterol and bile acid quantification

Cholesterol quantification of plasma was performed using a colorimetric HDL and LDL/VLDL cholesterol assay kit (Abcam). Whole plasma was first separated into HDL and VLDL/LDL fractions, after which they were quantified for levels of total cholesterol. Total bile acid in whole plasma was measured using a colorimetric total bile acids assay kit (Biovision). Cholesterol quantification of sebocyte cell lysate and media was performed using a total cholesterol kit (Biovision). Lipids were extracted from cell lysates by a modified folch method, and dried lipids were resuspended in assay buffer. Media was analyzed directly without a lipid extraction step. Final measurements were performed using a Tecan infinite M1000 Pro plate reader.

### Bile acid profiling

Plasma samples were diluted 1:9 in methanol and spiked with 2 ng labeled lithocholic acid (LCA) as the internal bile acid standard (CDN isotopes). Plasma samples were cleaned up by vacuum filtration through Phree Phospholipid Removal tubes (Phenomenex). The filtrate was then dried down using a centrifugal evaporator and resuspended in 20mM ammonium acetate buffer/ methanol (1/1). The samples were dissolved in 200 uL 1:1 methanol: 20mM ammonium acetate, and 30uL were placed in polypropylene autosampler vials for 20uL injection. The samples were analyzed by selected-reaction monitoring (SRM) on a Sciex 3200 QTRAP mass spectrometer equipped with Agilent 1100 HPLC. The column was a Waters BEH C18 1.7um particle, 2.1mm i.d. by 100mm long. The column was maintained at 50°C. The solvents used were A: 10mM ammonium acetate in water, pH 8; and B: 75% acetonitrile, 25% methanol. The flow rate was 200uL/min, and gradient elution took place starting at 20% B, ramping to 40% B at 30 minutes, then to 75% B at 45 minutes, holding at 75% B for 7 minutes, and returning to 20% B and re-equilibrating for 20 minutes. MS/MS transitions were monitored for all analyte bile acids with a dwell time of 100 milliseconds. The SRM transitions were divided into 4 windows based on the analytes eluting in each retention time window, to increase duty cycle and sensitivity. SRM transitions were also monitored for the spiked internal standard bile acids. All external bile acid standards were purchased from Steraloids. All samples were analyzed at UW-Madison Mass Spectrometry / Proteomics Facility.

### Statistics

Data are expressed as mean +/- S.E. with comparisons carried out using a two-sided Student’s t-test, using the program GraphPad Prism. p values <0.05 were considered significant.

## Results

### Skin-specific SCD1 deficient mice are lean, hyperphagic without steatorrhea

**Fig 1:**
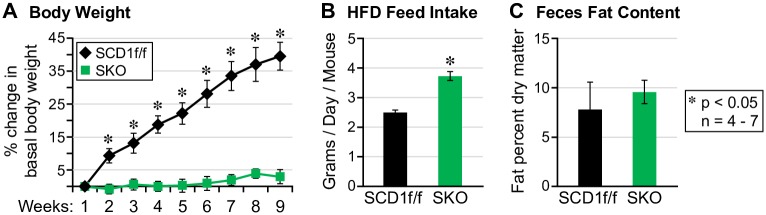
SKO mice are lean and hyperphagic on a high fat diet. (A) SKO mice are protected from weight gain on a high fat diet, (B) consume more food and (C) do not excrete more fat in feces.

As anticipated, SKO mice gained significantly less weight than controls despite consuming 1.5 times more HFD than controls (Fig 1A and 1B). Analysis of feces fat content was largely unchanged between both groups which suggests that increased excretion of fat-derived calories is not an explanation for their resistance to weight gain on a high fat diet (Fig 1C).

### Skin-specific SCD1 deficient mice exhibit enhanced cholesterol efflux from sebaceous glands to the surface of the skin

**Fig 2:**
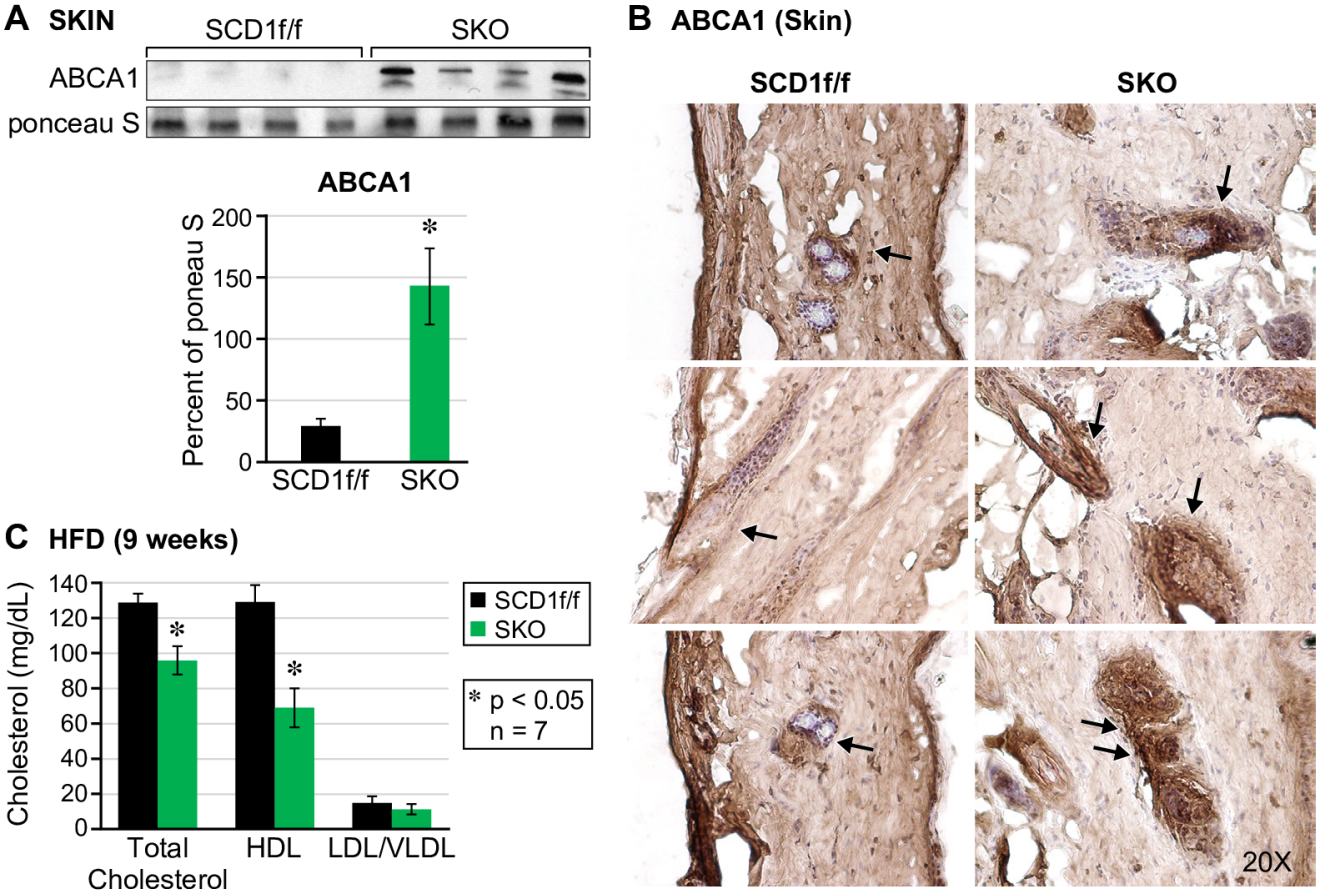
SKO mice demonstrate increased cholesterol efflux in whole skin, and decreased cholesterol levels in plasma. (A) Cholesterol transport protein ABCA1 is increased in the skin of SKO mice on a HFD diet. (B) ABCA1 is asymmetrically localized around hair follicles (C) Total, HDL and LDL cholesterol fractions are decreased in plasma of SKO mice on a high fat diet.

We previously reported that key cholesterol synthesis genes *Srebp2*, *Hmgcr* and *Sqle* are increased in the skin of chow-fed SKO mice [19]. In accordance with increased cholesterol synthesis in the skin, we also reported that skin surface lipids are enriched with free cholesterol [8]. In this study, we measured mRNA levels of cholesterol synthesis genes in the skin of HFD-fed SKO mice skin using rt-PCR (Part A of S1 Fig). Compared to our previous findings, HFD feeding decreased mRNA levels of cholesterol synthesis genes in the skin of SKO mice. Interestingly, levels of free cholesterol on the surface of SKO mice fed a HFD diet was still significantly elevated. (Part B of S1 Fig). To determine if levels of cholesterol on the surface of the skin are due to increased cholesterol efflux we measured protein levels of cholesterol transport protein ATP-binding cassette transporter (ABCA1). In HFD-fed mice, we found a sharp increase in ABCA1 protein expression in the skin which suggests that there is an upregulation of free cholesterol transport out of skin cells (Fig 2A). Histological analysis of ABCA1 expression revealed intense asymmetric staining around the hair follicles (Fig 2B), which is suggestive of sebaceous gland expression. Because skin is the largest organ of the body, we speculated that cholesterol efflux onto the surface of the skin might be enough to lead to decreased cholesterol in plasma. We found a significant decrease in all fractions of lipoprotein cholesterol in plasma (Fig 2B). Both HDL and LDL/VLDL cholesterol were decreased by 50% and 25% respectively, and total cholesterol levels decreased by 25%. We also show that SCD1 inhibition in SEB1 cells, a human sebocyte cell line, resulted in increased ABCA1 expression and cholesterol efflux. (Part C of S2 Fig). SCD1 inhibition caused intracellular cholesterol to decrease by 35% and extracellular cholesterol to increase by 8%. Cholesterol efflux in sebocytes is analogous to the whole-body movement of cholesterol from plasma onto the surface of SKO skin, and reaffirms the importance of SCD1-derived oleate for ACAT-mediated synthesis of cholesterol esters.

### Cyp7b1, a rate-limiting enzyme of bile acid synthesis, is increased in whole-skin of SKO mice

**Fig 3:**
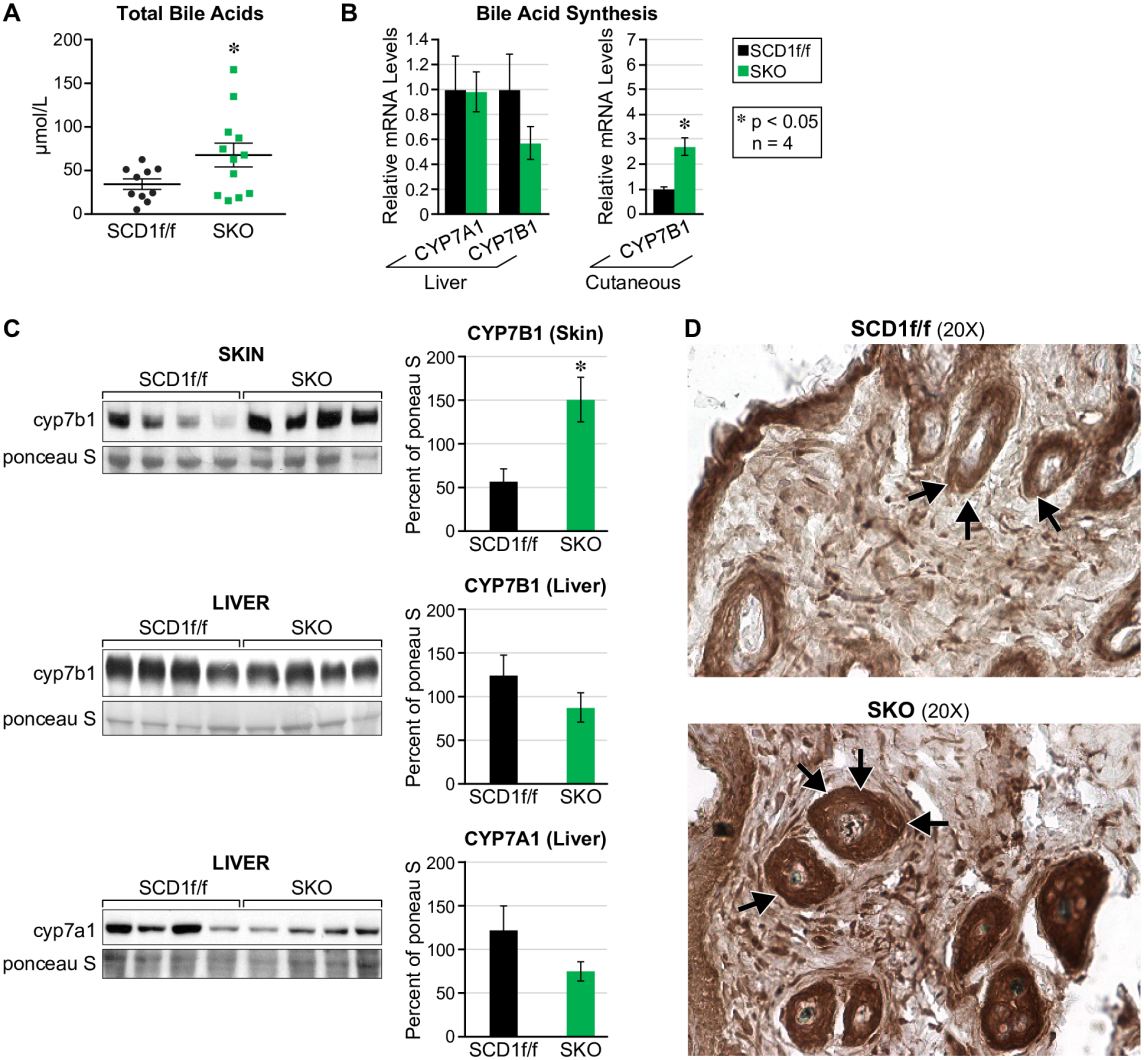
Cyp7b1, a rate-limiting enzyme of bile acid synthesis, is increased in whole-skin of SKO mice. (A) Total bile acids are increased by 50% in SKO mice relative to controls. (B) The rate-limiting enzymes for hepatic bile acid synthesis *cyp7a1* and *cyp7b1* are unchanged whereas *cyp7b1* in skin is increased. (C) Protein levels of cyp7a1 and cyp7b1 in liver and skin reflect mRNA levels. (D) Cyp7b1 protein expression is increased and localized around hair follicles.

Bile acids are the major product of cholesterol catabolism, and because of reduced plasma cholesterol, we speculated that in addition to cholesterol efflux through the skin, surplus skin-derived free cholesterol might also be metabolized into bile acids. As predicted, we found a 50% increase in total bile acids (Fig 3A). Surprisingly, rate-limiting bile acid synthesis genes in liver, *Cyp7a1 and Cyp7b1*, were unchanged, but *Cyp7b1* in the skin was increased 2.5-fold (Fig 3B) which suggests that the acidic pathway is upregulated in SKO mice. Additionally, protein levels of Cyp7a1 and Cyp7b1 in skin, and Cyp7a1 in liver reflected the genetic data, which further alludes to increased extrahepatic bile acid synthesis in SKO mice (Fig 3C). Furthermore, histological analysis of Cyp7b1 in whole skin revealed intense increased expression around hair follicles (Fig 3D).

### Tβ-MCA, a biomarker of extrahepatic bile acid synthesis, is increased in plasma of skin-specific SCD1 deficient mice

**Fig 4:**
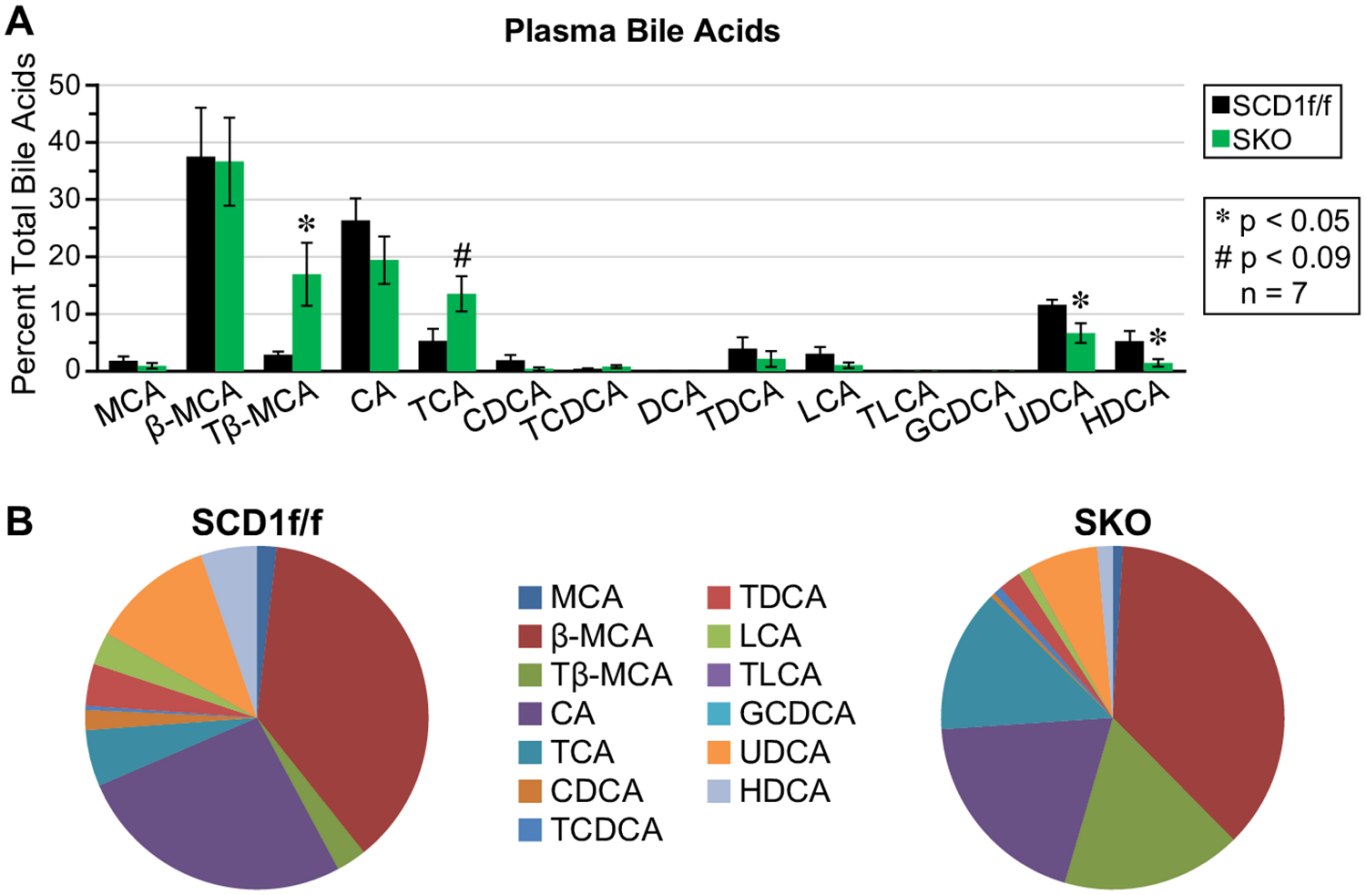
Bile acid composition is altered in SKO mice. (A) Taurine-conjugated bile acids are increased; Tβ-MCA, a marker of extrahepatic bile acid synthesis, is most significantly increased. Hydrophobic bile acids are decreased (B) A pie chart representation of bile acid composition.

The levels of primary bile acids that are produced by the acidic synthetic pathway differ from those that are produced by the neutral pathway. Specifically, levels of chenodeoxycholic acid (CDCA) in humans or β-muricholic acid (β-MCA) in mice are increased relative to cholic acid (CA) when the acidic pathway is increased [18]. Therefore, to test our hypothesis that SKO mice synthesize bile acids extrahepatically through increased Cyp7b1 expression in the skin, we measured plasma bile acids using liquid-chromatography mass spectrometry (LC-MS/MS). We prioritized analysis of free bile acids and their taurine conjugates because in rodents, taurine bile acid conjugates are more prevalent than glycine conjugates [15]. In fact, we demonstrate that in SKO mice, taurine conjugates are more prevalent than their free bile acid counterparts (Fig 4A and 4B). Taurine conjugation creates a more hydrophilic bile acid pool, and is associated with protection from metabolic disease. There were no significant changes in primary free bile acids between SKO and control mice. There was however, a significant decrease in secondary bile acids ursodeoxycholic acid (UDCA) and hyodeoxycholic acid (HDCA), indicating decreased gut microbial action on primary bile acids (Fig 4A and 4B).

### Enterohepatic circulation is decreased with skin-specific SCD1 deletion

**Fig 5:**
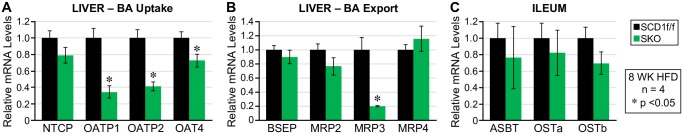
On a high fat diet, enterohepatic circulation of bile acids is downregulated. (A) Hepatic bile acid uptake and (B) export genes are downregulated and (c) bile acid transport genes are unchanged in the ileum.

Bile acids play a well-defined role in lipid digestion and absorption in the gut, and because of this, we measured expression of bile acid transport genes in the liver and in the ileum. In the liver, bile acid uptake genes *Ntcp*, *Oatp1*, *Oatp2 and Oatp4* were all decreased (Fig 5A) whereas bile acid export genes were either unchanged or decreased. *Bsep* and *Mrp2* which are expressed at the canalicular membrane of hepatocytes and function to export bile into the duodenum [20]. were unchanged or decreased respectively (Fig 5B). *Mrp3* and *Mrp4* which are expressed at the basolateral membrane of hepatocytes and function to export bile acids into plasma [20] were decreased or unchanged, respectively. And finally, bile acid transport genes of the ileum, *Asbt*, *Ostα* and *Ostß* are unchanged (Fig 5C). Overall, these data suggest that enterohepatic circulation is downregulated in SKO mice, which reinforces the concept that extrahepatic bile acid synthesis is important in the context of skin-specific SCD1 deficiency.

### Bile acid signaling is upregulated in brown adipose tissue of SKO mice

**Fig 6:**
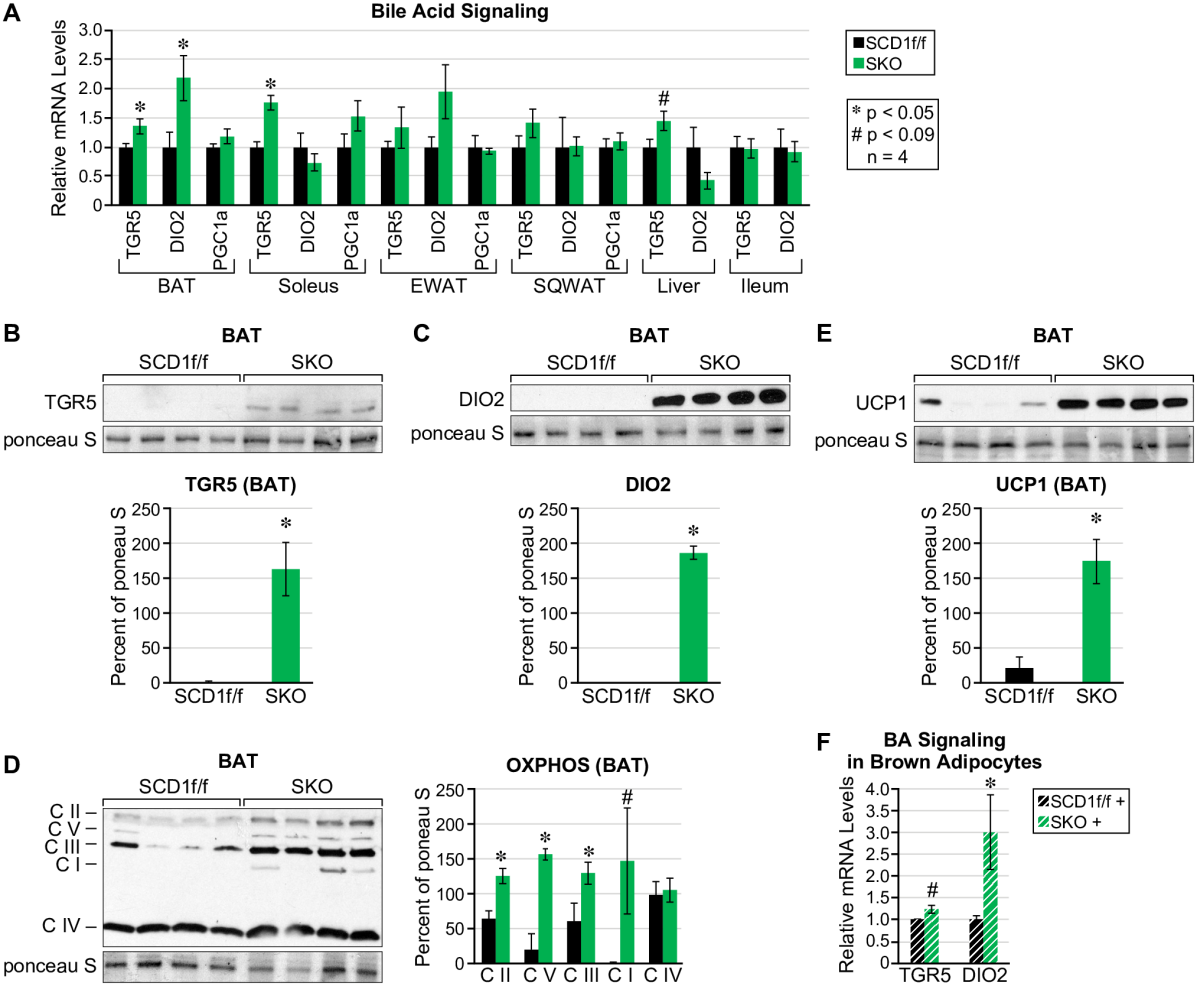
Bile acid-mediated thermogenesis is increased in SKO mice. (A) On a HFD, bile acid signaling via Tgr5 and Dio2 is upregulated at the level of mRNA in brown adipose tissue. (B) Protein levels of TGR5 and (C) DIO2 (D) UCP1 and (E) oxidative phosphorylation proteins CII, CIII and CV are also significantly increased in brown adipose tissue. (F) Bile acid signaling via Tgr5 and Dio2 is increased in wild-type brown adipocytes treated with SKO plasma.

To investigate the possibility that SKO mice employ bile acid mediated thermogenesis to increase energy expenditure, we measured expression of *Tgr5* and its downstream targets *Dio2* and *Pgc1a* [15] in brown adipose tissue (BAT), soleus muscle, epididymal and subcutaneous white adipose tissues, liver and ileum. We found that BAT and soleus muscle were the only tissues with a significant increase in *Tgr5*, whereas *Dio2* was significantly increased only in BAT (Fig 6A). We consequently followed-up with protein expression of TGR5 and DIO2 in brown adipose tissue and found that both TGR5 and DIO2 were significantly increased in SKO mice (Fig 6B and 6C). Because increased oxidative phosphorylation mediates increased energy expenditure, we measured protein levels of mitochondrial proteins, complex I through V and found that complexes II, III, and V were significantly upregulated (Fig 6D). We also measured levels of UCP1 and found a sharp increase in protein expression (Fig 6E). To confirm that bile acids in SKO plasma leads to increased expression of TGR5 and DIO2, we treated wild-type brown adipocytes with mouse plasma from SCD1f/f or SKO mice, and found that mRNA levels of *Tgr5* and *Dio2* were trending or significantly increased, respectively (Fig 6F).

## Discussion

In this study, we identified a role for bile acids in the lean and metabolically desirable phenotype of SKO mice. Because of the shortage of endogenously synthesized oleate in SKO skin, a surplus of free cholesterol accumulates. Some of this cholesterol is effluxed through ABCA1 onto the surface of the skin as an immediate response to increased intracellular cholesterol. Because ABCA1 expression is increased in SCD1-inhibited sebocytes, cholesterol secretion onto the surface of SKO skin could be mediated by holocrine secretion of cholesterol-rich sebum. Alternatively, free cholesterol can be neutralized by conversion into other molecules including oxysterols, bile acids, vitamin D and steroid hormones. We focused on bile acids because mice overexpressing the bile acid-specific receptor TGR5 display increased energy expenditure, decreased hepatic steatosis, and are resistant to diet-induced obesity [21]. High levels of Tβ-MCA in plasma of SKO mice in addition to increased levels ofcyp7b1 in skin, and decreased levels of cyp7a1 in the liver, suggests upregulation of extrahepatic bile acid synthesis. Unchanged or decreased expression of genes involved in enterohepatic circulation further allude to the importance of extrahepatic bile acid synthesis in SKO mice. Furthermore, we observed that the bile acid pool of SKO mice is more hydrophilic which is known to be protective against the development of metabolic disease [22].

Tβ-MCA has also been identified as a marker of metabolic health in mice [23]. And finally, we confirmed upregulation of TGR5 and DIO2 expression in BAT of SKO mice as well as brown adipocytes treated with their plasma.

Since the discovery of TGR5 in 2002, there have been many studies on its protective role in obesity, diabetes, NAFLD and inflammation [15,24,25]. Similar to skin-specific SCD1 deficiency, TGR5 activation leads to increased energy expenditure, and protection from hepatic steatosis and weight gain [21]. Curiously, in SKO mice, bile acids that are known to potently activate TGR5, such as HDCA, LCA, and DCA [15] are decreased. However, the bile acids that are significantly increased in SKO mice, Tβ-MCA and TCA, can still bind to and signal through TGR5 [15]. Importantly, taurine conjugated bile acids activate TGR5 more potently than their glycine conjugated and unconjugated counterparts [15]. It is also known that bile acids upregulate expression of TGR5 which further facilitates bile acid signaling [26]. Furthermore, the hormone response element for the nuclear bile acid receptor farnesoid X receptor (FXR) has been recently identified in the promoter region of the TGR5 gene [26]. This is noteworthy because Tβ-MCA is a known potent FXR agonist, and it may play an as yet identified role in TGR5 regulation [15,25]. Tβ-MCA also inhibits transcription of *Cyp7a1* via FXR which is in agreement with decreased levels of *Cyp7a1* in SKO livers [15]. Because we demonstrate increased expression of TGR5 and DIO2 in both brown adipose tissue, and brown adipocytes treated with SKO plasma, we suggest that TGR5 plays a role in the increased energy expenditure displayed by SKO mice. Identifying this path for increased energy expenditure is especially significant, since we have previously ruled out increased cold perception as the cause of increased energy expenditure of SKO mice [19]. Our first experiment demonstrated that SKO mice on a HFD were protected from hypothermia at 4°C indicating that they are not susceptible to the cold while on a HFD diet [8]. And our second study indicated that SKO mice at thermoneutrality (33°C) were still protected from weight gain [19], indicating that the lean SKO phenotype is not due to increased fuel consumption for heat generation.

Extrahepatic bile acid synthesis is not a new concept; cyp7b1 is expressed in many tissues and is important for supplementing bile acid production during liver disease [18]. Also, cyp7b1 has recently been identified in human lungs as a biomarker for the development of pulmonary arterial hypertension [27]. The acidic pathway however is not widely believed to be independently capable of completely synthesizing bile acids, because of a lack of liver-specific enzymes [18]. That is, oxysterol intermediates must subsequently travel to the liver for completion of bile acid synthesis [18]. Nevertheless, a 2009 publication indicated the presence of all major enzymes of the neutral bile acid synthesis pathway in human ovaries [28].

Because of the low blood cholesterol levels displayed by SKO mice, future studies are needed to determine if SCD1 deletion in the skin protects mice from the development of atherosclerotic plaques. Previous studies suggest that liver specific or whole-body SCD1 deletion might be detrimental to cardiovascular health. Global SCD1 knock-out mice that were crossed with low density lipoprotein receptor (LDLR)-deficient mice displayed increased atherosclerosis [29]. And SCD1 ASO treatment that inhibits SCD1 in the liver and white adipose tissue also led to increased atherosclerosis [30]. In the latter study, SCD1 function was intact in the skin, and because of this, we suggest that SCD1 deficiency in the liver worsens cardiovascular health, but SCD1 deletion in the skin could be protective. We speculate that this is because liver SCD1 deficiency leads to increased cholesterol efflux from the liver into circulation, whereas skin SCD1 deficiency leads to increased cholesterol efflux from within skin unto the surface of the skin. Furthermore, increased TGR5 activation through macrophages is a known cardio-protective phenotype, because TGR5 activated macrophages are less likely to take up oxidized LDL particles and develop into foam cells [15]. One potential study is to cross SKO mice with an atherosclerotic mouse model, such as the LDLR-deficient mouse, to determine if SCD1 deletion in the skin offers protection from developing atherosclerotic lesions. If skin-specific SCD1 deficiency is indeed cardio-protective, then in addition to providing protection from obesity, SCD1 in the skin may be an as yet discovered target for a cholesterol-lowering compound.

## Supporting information

S1 Fig On a chow diet: (A) SREBP2 is unchanged but target genes Hmgcr and Sqle are significantly increased in skin, whereas, on a HFD these genes are insignificantly different. (B) Free cholesterol extracted from the dorsal skin of SKO mice on a HFD is increased 4-fold in SKO mice. Sterol regulatory element-binding protein 2 (Srebp2), HMG-CoA reductase (Hmgcr) and squalene epoxidase (Sqle).
(DOCX)

S2 Fig (A) SCD1 inhibition is confirmed in SEB1 cells: MUFAs are decreased and SFAs are increased. (B) With SCD1 inhibition, intracellular levels of cholesterol are decreased and extracellular levels are increased. (C) In sebocytes, ABCA1 expression is increased with treatment of A939572. P: Presebocytes, C: Control, D: DMSO S: SCD1 inhibitor (A939572).
(DOCX)

S1 Supporting Methods (DOCX)
