# Supplementary figures and images for: Increased hydrophilic plasma bile acids are correlated with protection from adiposity in skin-specific stearoyl-CoA desaturase-1 deficient mice
Source: PLoS One. 2018 Jul 2;13(7):e0199682. doi: 10.1371/journal.pone.0199682 (PMC6028101; doi:10.1371/journal.pone.0199682)

S1 Fig

A

**
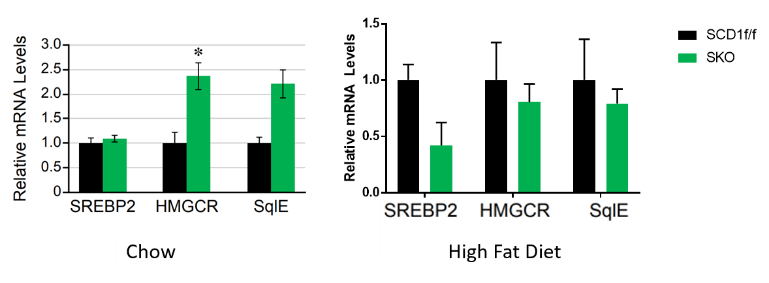
**

|  |
| --- |
|  |

B

Supplement: S1 Fig — On a chow diet: (A) SREBP2 is unchanged but target genes Hmgcr and Sqle are significantly increased in skin, whereas, on a HFD these genes are insignificantly different. (B) Free cholesterol extracted from the dorsal skin of SKO mice on a HFD is increased 4-fold in SKO mice. Sterol regulatory element-binding protein 2 (Srebp2), HMG-CoA reductase (Hmgcr) and squalene epoxidase (Sqle). (DOCX) [file pone.0199682.s001.docx]

S2 Fig

**A**


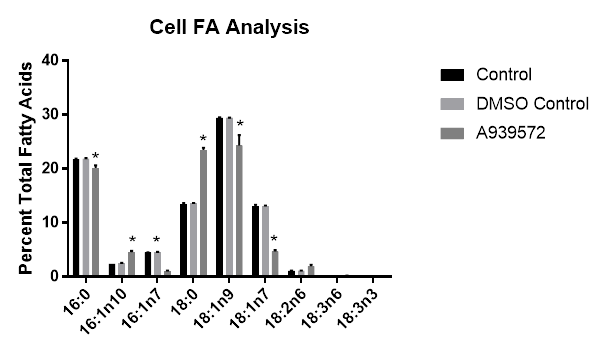


**B**


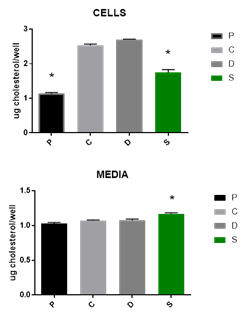


C


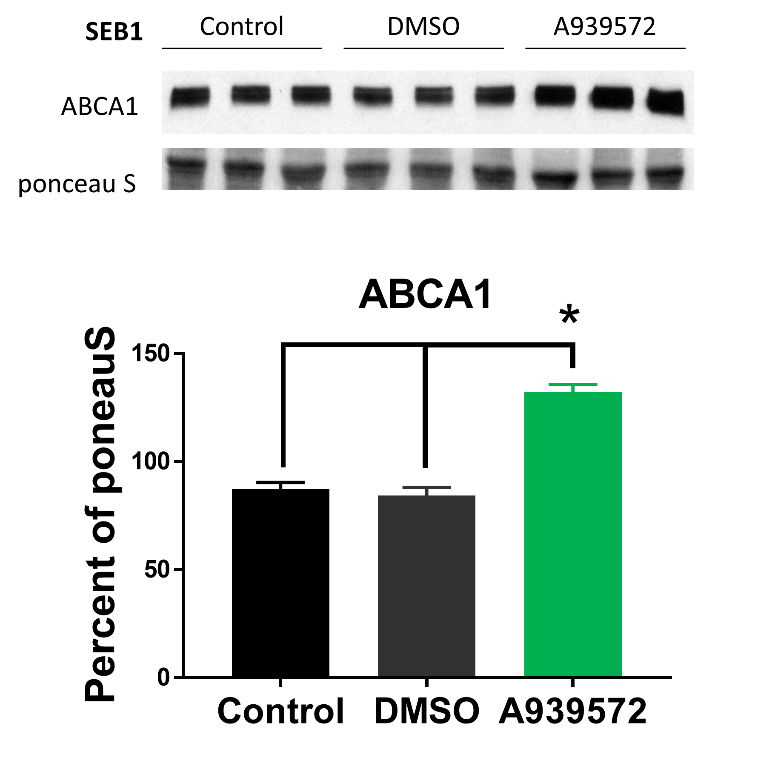

Supplement: S2 Fig — (A) SCD1 inhibition is confirmed in SEB1 cells: MUFAs are decreased and SFAs are increased. (B) With SCD1 inhibition, intracellular levels of cholesterol are decreased and extracellular levels are increased. (C) In sebocytes, ABCA1 expression is increased with treatment of A939572. P: Presebocytes, C: Control, D: DMSO S: SCD1 inhibitor (A939572). (DOCX) [file pone.0199682.s002.docx]
