# Supplementary material for: Increased hydrophilic plasma bile acids are correlated with protection from adiposity in skin-specific stearoyl-CoA desaturase-1 deficient mice
Source: PLoS One. 2018 Jul 2;13(7):e0199682. doi: 10.1371/journal.pone.0199682 (PMC6028101; doi:10.1371/journal.pone.0199682)
Supplement: S1 Supporting Methods — (DOCX) [file pone.0199682.s003.docx]

**S1 Supporting Methods:** Lipid extraction and gas chromatography of SEB1 cells.

Cell pellet lipids were extracted following a modified Folch method. Heptadecanoic acid was added as an internal control for fatty acid quantification, and pentadecanoic acid was added as an internal control of transmethylation efficiency. Lipids were extracted and transmethylated using boron trifluoride in 14% methanol (Sigma). Fatty acid methyl esters were suspended in hexane and analyzed by gas chromatography (GC). Chromatograms were analyzed using HP ChemStation software. Results were calculated to express fatty acid composition as a percent of total lipids.

**Diet Composition Links**

Research Diets

D12492: https://researchdiets.com/formulas/d12492

Purina

5015: http://www.labdiet.com/cs/groups/lolweb/@labdiet/documents/web_content/mdrf/mdi4/~edisp/ducm04_028439.pdf
